# Study protocol for an adaptive, multi-arm, multi-stage (MAMS) randomised controlled trial of brief remotely delivered psychosocial interventions for people with serious mental health problems who have experienced a recent suicidal crisis: Remote Approaches to Psychosocial Intervention Delivery (RAPID)

**DOI:** 10.1186/s13063-024-08293-5

**Published:** 2024-07-06

**Authors:** Melissa Pyle, Lucy Loftus, Richard Emsley, Daniel Freeman, Steven Gillard, Andrew Gumley, Justyna Sierpatowska, Lisa Wood, Rory C. O’Connor, Paul Pfeiffer, Sharon Anne Simpson, Nicole Cockayne, Gemma Shields, Ariane Beckley, Helen Beckwith, Maria Filippidou, Callum Glen, Stephanie Allan, Raj Hazzard, Eleanor Longden, Heather Peel, Mark Larsen, Sandra Bucci, Anthony P. Morrison

**Affiliations:** 1 The Psychosis Research Unit, Department of Psychology, Greater Manchester Mental Health NHS Foundation Trust, Manchester, UK; 2 Department of Biostatistics & Health Informatics, Institute of Psychiatry, Psychology & Neuroscience, King’s College London, London, UK; 3 Department of Experimental Psychology, Medical Sciences Division, University of Oxford, Oxford, UK; 4 Oxford Health NHS Foundation Trust, Oxford, UK; 5 School of Health & Psychological Sciences, City, University of London, London, UK; 6 School of Health and Wellbeing, University of Glasgow, Glasgow, UK; 7 East London NHS Foundation Trust, London, UK; 8 Division of Psychiatry, University College London, London, UK; 9 Research and Development, Northeast London NHS Foundation Trust, Essex, UK; 10 Department of Psychiatry, University of Michigan Medical School, Ann Arbor, MI USA; 11 VA Center for Clinical Management Research, Ann Arbor, MI USA; 12 Black Dog Institute, University of New South Wales, Sydney, Australia; 13 Manchester Centre for Health Economics, Division of Population Health, School of Health Sciences, University of Manchester, Manchester, UK; 14 McPin Foundation, 7-14 Great Dover Street, London, UK; 15 Division of Psychology and Mental Health, University of Manchester, Manchester, UK; 16 Centre for Big Data Research in Health, University of New South Wales, Sydney, Australia

**Keywords:** Serious mental health problems, Suicide, Psychiatric admission, Psychosocial intervention, Adaptive trial design, Multi-arm, Multi-stage, Randomised controlled trial

## Abstract

**Background:**

People with serious mental health problems (SMHP) are more likely to be admitted to psychiatric hospital following contact with crisis services. Admissions can have significant personal costs, be traumatic and are the most expensive form of mental health care. There is an urgent need for treatments to reduce suicidal thoughts and behaviours and reduce avoidable psychiatric admissions.

**Methods:**

A multi-stage, multi-arm (MAMS) randomised controlled trial (RCT) with four arms conducted over two stages to determine the clinical and cost effectiveness of three psychosocial treatments, compared to treatment as usual (TAU), for people with SMHP who have had recent suicidal crisis. Primary outcome is any psychiatric hospital admissions over a 6-month period. We will assess the impact on suicidal thoughts and behaviour, hope, recovery, anxiety and depression. The remote treatments delivered over 3 months are structured peer support (PREVAIL); a safety planning approach (SAFETEL) delivered by assistant psychologists; and a CBT-based suicide prevention app accessed via a smartphone (BrighterSide). Recruitment is at five UK sites. Stage 1 includes an internal pilot with a priori progression criteria. In stage 1, the randomisation ratio was 1:1:1:2 in favour of TAU. This has been amended to 2:2:3 in favour of TAU following an unplanned change to remove the BrighterSide arm following the release of efficacy data from an independent RCT. Randomisation is via an independent remote web-based randomisation system using randomly permuted blocks, stratified by site. An interim analysis will be performed using data from the first 385 participants from PREVAIL, SAFETEL and TAU with outcome data at 6 months. If one arm is dropped for lack of benefit in stage 2, the allocation ratio of future participants will be 1:1. The expected total sample size is 1064 participants (1118 inclusive of BrighterSide participants).

**Discussion:**

There is a need for evidence-based interventions to reduce psychiatric admissions, via reduction of suicidality. Our focus on remote delivery of established brief psychosocial interventions, utilisation of different modalities of delivery that can provide sustainable and scalable solutions, which are also suitable for a pandemic or national crisis context, will significantly advance treatment options.

**Trial registration:**

ISRCTN33079589. Registered on June 20, 2022.

**Supplementary Information:**

The online version contains supplementary material available at 10.1186/s13063-024-08293-5.

## Introduction

### Background and rationale

In the United Kingdom (UK), psychiatric inpatient care has seen a sustained rise following the COVID-19 pandemic and from increased socio-economic deprivation [1]. In England, a compulsory admission under the Mental Health Act 2007 (formerly Mental Health Act 1983) is mandatory in cases of considerable danger to self, including suicidal ideation [2]. The rates of compulsory admissions have continued to increase each year in the UK [3]. Yet, psychiatric hospital admissions (PHAs) can have significant personal costs such as stigma, loss of relationships, employment and housing, and traumatisation [4, 5]. PHAs are the most expensive form of mental health care with the estimated costs of involuntary psychiatric admissions in the UK totalling £6.8 billion [6]. A psychiatric admission is often seen as an unacceptable form of care to patients as it represents a restriction on personal freedom and wards are not always safe, therapeutic or conducive to recovery [4]. The UK National Health Service (NHS) acknowledges that significant improvement is required to ensure inpatient settings are therapeutic [7].

Patients with serious mental health problems (SMHPs) have an elevated risk of physical health comorbidities, i.e. cardiovascular and metabolic health conditions [8], and the COVID-19 pandemic introduced a new challenge for vulnerable patients and service providers due to the increased risk of transmission in a ward environment [9]. There is already a large and discriminatory mortality gap for those with an SMHP diagnosis, with those who experience psychosis, on average, living 15 years less in comparison to people without a psychosis diagnosis [10]. Such elevated risk in the context of an epidemic or pandemic for those who are a psychiatric inpatient is of concern.

Crisis teams are part of a UK strategy to provide community care to people with SMHPs who are experiencing a suicidal crisis with the aim of reducing PHAs [11]. However, more than half of the crisis team patients have an admission within a year of discharge from hospital [12]. People with an SMHP diagnosis, such as psychosis, bipolar and emotionally unstable personality disorder (EUPD), are the diagnostic groups most likely to have an admission following contact with a crisis team [13] and the majority of first admissions are due to suicidality [14]. Strategies to reduce PHAs include crisis planning, self/symptom management, relapse prevention and monitoring of suicidal risk [15]. Of these, interventions that focus on symptom management, relapse prevention and patient functioning can reduce the average number of readmissions [16]. However, the methodological limitations to existing studies, including risk of bias and inconsistent results, suggest that a high-quality trial of self-management strategies with admissions as the primary outcomes is required [16].

It is a deeply concerning fact that one in 100 deaths are by suicide and that over 700,000 people die per year because of suicide [17]. This has far-reaching emotional, social and economic impacts and it is crucial to have an evidence base for interventions that support people in a crisis to prevent suicide. Interventions that are designed to reduce suicidal ideation and behaviour may have also have an indirect effect on reducing admissions given the majority of first admissions occur in response to a suicidal crisis [14].

A recent network meta-analysis reports that dialectical behaviour therapy (DBT), brief intervention and contact (BIC) and cognitive therapy (CT) were superior when compared to treatment as usual (TAU) in preventing suicide attempts [18]. Whilst these interventions show promise to reduce suicide attempts, the authors conclude that further research is needed to validate which interventions are most effective and to our knowledge it is unclear whether there is an indirect effect on PHAs. One brief intervention and contact approach delivered remotely, with telephone follow-up support, is SAFETEL [19]. This intervention incorporates a safety planning approach with identification of warning signs, distraction techniques and social/professional support sources with follow-up telephone support and has its origins in CT for suicidality. This approach has been shown to be both safe and acceptable for people in the UK who have presented to emergency departments after a suicide attempt [20]. Digital interventions have been proposed to improve access to treatment for people who are at risk of suicide and have the potential to extend the scalability and accessibility of an intervention [21]. A review of the evidence regarding digital cognitive behavioural therapy (CBT) showed that those who received digital CBT had a significant reduction on suicidal ideation scores in comparison to a control group, but did not show a significant effect for suicide attempts and that human support with completion was required to increase engagement with a digital intervention [22]. One digital CBT intervention included in the review which showed initial promise for reducing suicidal thinking is an online self-guided programme for adults designed to help reduce suicidal ideation (Living with Deadly Thoughts: LwDT) [23]. LwDT was further developed from a web-based intervention into a smartphone app version to improve access (BrighterSide smartphone app) with considerable input from people with lived experience of suicidal thinking and behaviours [24]. One brief, psychosocial approach that incorporates strategies derived from BIC and motivational interviewing with peer support (PS) is PREVAIL (Peers for Valued Living), which has demonstrated feasibility and acceptability for patients at high risk of suicide [25]. PS is highly valued by service users, forming part of the vision for the future of mental health services [26] and is associated with positive effects on measures of hope [27], which protects against suicidal ideation [28]. As outlined, these three candidate interventions have demonstrated feasibility and acceptability and are deliverable remotely, which may increase scalability if effective and future proof intervention delivery in the context of an epidemic, pandemic or other crises.

In summary, there is a need to prevent avoidable PHAs for people who have SMHP. There is strong theoretical evidence to suggest that interventions which target suicidal ideation and behaviour can reduce avoidable admissions, since many admissions are because of a suicidal crisis. The Remote Approaches to Psychosocial Intervention Delivery (RAPID) trial builds on the feasibility and acceptability data for PREVAIL, SAFETEL and BrighterSide. As a multi-arm, multi-stage (MAMS) trial, RAPID uses an adaptive design that allows for these multiple interventions to be evaluated within one trial with pre-planned adaptations that allow for early selection of interventions that are promising, or the removal of interventions that are shown to be futile [29]. As such, the RAPID trial is an efficient approach to establishing the evidence base regarding the effectiveness of these interventions, within one single study, in comparison to a traditional randomised controlled trial (RCT) design [29]. Our focus on remote delivery of these established brief psychosocial interventions, if effective, would provide evidence for interventions that are sustainable and scalable solutions.

## Objectives

Our primary objective is to answer the question of which brief, remote psychosocial intervention for people with SMHPs who report recent suicidal ideation, or a suicide attempt is most clinically effective and cost-effective in preventing avoidable PHAs in comparison to TAU, and to determine the safety of the interventions.

Specific hypotheses are that, compared to TAU, our brief, remote interventions plus TAU will lead to:

1. Reduction in psychiatric hospital admissions over 6 months (primary outcome)
2. Reduction in psychiatric hospital admissions over 3 months
3. Reduction in suicidal ideation over 3 and 6 months
4. Improvement in user-defined recovery and quality of life over 3 and 6 months

We also hypothesise that our interventions will be cost-effective over 6 months in comparison to TAU.

## Trial design

The original RAPID study design was a four-arm, superiority, randomised controlled trial (RCT) with a MAMS adaptive design with three candidate interventions (PREVAIL, SAFETEL and BrighterSide) included in stage 1 with a randomisation allocation ratio of 1:1:1:2 favouring TAU. A decision was made in April 2023 to remove the BrighterSide arm (see ‘Unplanned trial adaptations’ section below), resulting in a new treatment allocation of 2:2:3 favouring TAU. The pre-planned trial adaptations are an interim analysis on the primary outcome of any psychiatric hospital over 6 months using data from the first 385 PREVAIL, SAFETEL and TAU participants recruited with outcome data at the 6-month follow-up.

### Unplanned trial adaptations

Several important changes to the design occurred after the trial commenced, which were unplanned changes to the design. In line with the Adaptive Designs CONSORT Extension (ACE) statement [30], we report the unplanned changes in this protocol. All changes were approved by the funder, and where regulation was required, was approved by the Medicines and Healthcare Products Regulatory Agency (MHRA), the UK Research Ethics Committee (REC) and Health Research Authority (HRA).

In response to feedback from NHS crisis teams regarding the duration of care, the inclusion criteria was changed from ‘*Receiving care from a HBTT (referrals to HBTT Team are associated with increased risk of a psychiatric hospital admission in the near future)*’ to ‘*Currently receiving care from a Home-Based Treatment Team/Crisis team or have done so within the last 14 days, since referrals to HBTT/Crisis Team are associated with increased risk of a psychiatric hospital admission in the near future*’. In response to feedback from crisis teams regarding the patient population diagnoses, we was advised to include patients with a post-traumatic stress disorder (PTSD) or complex PTSD (cPTSD) diagnosis. As such the inclusion was changed to include patients with these diagnoses to ensure generalisability of the study findings to the crisis team patient population.

Since the initial development of this protocol, the BrighterSide smartphone app has been evaluated in a large study of 550 participants from the general population who reported suicidal ideation and results showed there was no significant improvement for the primary outcome of suicidal ideation in comparison to the waitlist control group [24]. In response to the new scientific data, the RAPID design was changed from a four-arm trial to a three-arm trial following removal of the BrighterSide intervention arm. The decision was made in conjunction with the RAPID trial Data Monitoring Committee (DMC), Trial Steering Committee (TSC), the study sponsor and the funder. The following changes were made to the design:

i. Randomisation ratio changed from 1:1:1:2 favouring TAU to 2:2:3 favouring TAU.
ii. Target sample size changed from 1235 to 1064 (not inclusive of the 54 participants randomised to BrighterSide at the time of dropping this arm) with no loss of power.
iii. Number required for the interim analysis will change from the first 559 participants recruited with available primary outcome data to first 385 PREVAIL, SAFETEL and TAU participants with primary outcome data and provide 92% power.
iv. Removal of the following exclusion criterion since it was relevant only to the BrighterSide app, ‘*visual impairment, severe enough to prevent engagement with the BrighterSide app as provision would be impossible on both financial and logistical ground*’.
v. The RAPID trial was initially classified as a clinical trial of a medical device by the MHRA based on the inclusion of the BrighterSide smartphone app. Following removal of this intervention and on exit of the last participant allocated to BrighterSide in November 2023, the trial ceased to be a clinical trial of a medical device and under the regulation of the MHRA.

All participants already recruited to the BrighterSide arm were followed up to 6-month follow-up and completed involvement as per the study protocol.

Our trial included an internal pilot. Following review of the internal pilot recruitment data by the funder in August 2023, the monthly recruitment target was reduced from 50 to 35 participants per month effective from June 1, 2023.

## Methods: participants, interventions and outcomes

### Study setting

RAPID is conducted in UK NHS crisis services, which are typically referred to as Home-Based Treatment Teams (HBTT) across five locations in England and Scotland. The five settings are East London NHS Foundation Trust, NHS Greater Glasgow and Clyde, Greater Manchester Mental Health NHS Foundation Trust, Northeast London NHS Foundation Trust and Oxford Health NHS Foundation Trust. Participant assessments take place in community settings including the home of the participant or at a community clinic. All interventions are delivered remotely via videoconference or by telephone. Full details of the study sites can be found at our ISRCTN record: 10.1186/ISRCTN33079589. We use SPIRIT reporting guidelines for this protocol [31].

### Eligibility criteria

Our inclusion criteria are as follows:

1. Currently receiving care from a Home-Based Treatment Team/crisis team or have done so within the last 14 days, since referrals to HBTT/crisis team are associated with increased risk of a psychiatric hospital admission in the near future
2. Aged 16+
3. Meet criteria for a diagnosis of SMHP (schizophrenia spectrum, bipolar, major depressive disorder, emotionally unstable personality disorder (EUPD), PTSD or cPTSD) since these diagnoses account for the majority of PHAs for mental health difficulties
4. Experienced suicidal ideation or attempt within the last month/current crisis episode, as operationalised by answering ‘yes’ to items 1 or 2 of the Columbia-Suicide Severity Rating Scale
5. Able to provide informed consent
6. Receiving care from a Community Mental Health Team or Early Intervention Service, to ensure ongoing specialist mental health support following discharge from HBTT

Our exclusion criteria are as follows:

1. Organic impairment, as this could be the cause of mental health symptoms rather than a SMHP.
2. Non-English speaking, since two of the interventions are remotely delivered talking therapies and one of the interventions is a smartphone app which has only been developed in English. Provision for non-English speakers would be impossible on both financial and logistical grounds.
3. Primary diagnosis of a drug or alcohol dependence, as this could be the cause of mental health symptoms rather than a SMHP.
4. Moderate to severe learning disability as confirmed by the participant’s responsible clinician in their care team.
5. For both ethical and safety reasons, immediate risk to others as confirmed by the participant’s responsible clinician in their care team.
6. Currently receiving psychiatric inpatient care (since people in recent contact with crisis teams may have already been admitted to hospital).

### Who will take informed consent?

Informed consent is obtained from potential trial participants by Research Assistants (RA) at each of the five sites. RAs are trained in Good Clinical Practice (GCP) and are supervised by the site Principal Investigators. Potential participants are provided with a REC-approved Participant Information Sheet (PIS) and are given at least 24 h to consider the information before providing informed consent. We prioritise recording informed consent in writing via a wet-ink signature from both the participant and the researcher. However, where it is not feasible to seek a wet-ink signature from the participant on paper forms, i.e. when a potential participant is extremely clinically vulnerable to COVID-19 and risks of meeting in-person are significant, informed consent is taken via a remote method (telephone or videoconference). As approved by the study sponsor, the REC and HRA, the consent visit is audio recorded and the audio recording is retained in the Trial Master File as evidence of the informed consent process. For audio-recorded consent, the participant is required to state their full name and date of consent, the researcher reads out each statement on the consent form and in return the potential participant verbally confirms their agreement with each statement of consent, the researcher adds their wet-ink signature to the consent form as evidence of their taking consent and where possible, a wet-ink signature is sought from a participant at a later date. All participants are provided with a copy of their consent form.

## Interventions

### Explanation for the choice of comparators

The control condition is TAU, consisting of multi-disciplinary care delivered by crisis teams. Different psychosocial interventions are recommended in National Institute for Health and Care Excellence guidelines for the different diagnostic groups, so there is no single active comparator that would be suitable. TAU services are not asked to withhold any treatment and all routine or additional treatments are monitored via the Economic Patient Questionnaire, which tracks access to health and social care services.

### Intervention description

#### SAFETEL

SAFETEL is a brief, psychosocial intervention delivered by Assistant Psychologists (APs) employed by the NHS and delivered by telephone or videoconference [19, 20]. Participants allocated to the SAFETEL intervention are offered up to 12 sessions of the intervention over a 3-month intervention period. In the initial phase of delivery, a safety plan is developed collaboratively. It has six components: (i) identifying warning signs of an impending suicidal crisis; (ii) utilising internal coping strategies; (iii) engaging social contacts and social settings to distract from suicidal thoughts; (iv) contacting social supports for assistance in resolving the suicidal crisis; (v) contacting mental health professionals; (vi) minimising access to lethal means. The suitability, barriers and likelihood of employing these strategies during a suicidal crisis are explored, as well as examples of such strategies being provided. Follow-up contacts are provided over a period of 3 months. The follow-up calls comprise three components: (1) suicide risk assessment and mood check; (2) review of the participant’s safety plan, with revisions made if required; (3) supporting treatment engagement through exploration of barriers to engagement, motivational enhancement, problem-solving and support. The core element of SAFETEL is the collaborative development of the safety plan (within the 1st session). This is a prioritised list of coping strategies and supports that individuals can use during or preceding suicidal crises. It has been developed to also address challenges in continuity of care across vulnerable transitions. SAFETEL incorporates telephone follow-up to conduct periodic risk assessment and mood checks. This allows for the continuous review of the safety plan and provides opportunities to problem-solve obstacles to treatment and help with linkage to services, if necessary. It actively incorporates evidence-based suicide prevention strategies, including facilitation of problem solving and coping skills, identification and use of social supports and emergency contacts, lethal means restriction, service linkage and motivational enhancement to promote community treatment engagement.

#### PREVAIL

PREVAIL is a brief, psychosocial intervention delivered by Peer Support Workers (PSWs) employed by the NHS and is delivered by telephone or videoconference [25]. Participants allocated to the PREVAIL intervention are offered up to 12 sessions of the intervention over a 3-month intervention period. The key components of the PREVAIL intervention include common elements of peer support (PS) such as supportive listening and PSW sharing recovery stories, improving hope and belongingness, managing acute suicide risk including safety planning, maintaining both peer and PSW wellness and principles of motivational interviewing. PREVAIL is delivered in three phases: (i) assessment and getting to know you, (ii) active involvement and (iii) ending and consolidation.

Semi-structured conversations incorporate suicide prevention strategies derived from CBT and motivational interviewing (MI), including goal setting, distress tolerance, and increasing optimism and social connectedness. These conversations use a standardised format including the steps of Invite, Learn, Share and Motivate. In the Invite step, permission is sought from the participant to have a conversation about a hope or belongingness-related topic. During the Learn step, information regarding what the participant has already tried and what the participant thinks might be helpful or relevant to their situation is elicited. During the Share step, helpful suggestions are made based on the PSW’s personal experience or knowledge. The Motivate step engages the participant in ‘change talk’, including how acting might be helpful to them and how they might implement changes.

The final phase, focusing on endings, consolidation and future directions (including future access to peer support), will span sessions 11–12 (approximately), although endings and the time-limited nature of PREVAIL will be regularly discussed throughout all phases. The PSW may transition to a step-down phase at session 10 by moving to fortnightly contact. The aim of the final sessions is to consolidate learning, review what has been helpful and develop a plan to maintain gains. The format of delivery will be flexible throughout the 12 sessions.

The PS values of shared lived experience; reciprocity and mutuality; validating experiential knowledge; choice and control; discovering strengths and making connections are core to delivery. It is expected that peer relationships will offer emotional and instrumental support and promote hope through role modelling. PSWs will offer validation of the person’s suicidal experiences and concerns by showing understanding through their own experiences and enabling the participant to engage in talking about their experiences and concerns relating to suicide and hopes for the future.

#### BrighterSide smartphone app

BrighterSide is a self-guided smartphone app to help those with suicidal thinking to understand their thoughts and develop the best skills and strategies to help manage them [24]. The app contains five modules that are primarily based on CBT and DBT. The module topics are (1) Understand Your Thoughts; (2) Prevent a Crisis; (3) Navigate your Emotions; (4) Navigate Your Thoughts; and (5) Plan for the Future. BrighterSide allows users to personalise their experience with the following key features: develop a personalised safety plan, daily check-ins to help users connect with their feelings (this option can be skipped), coping tools including claiming and distracting techniques, pathway selection through the app based on the user’s preference and favourites to allow users to save their favourite activities and content. Content is not time-gated, with all five modules available immediately on installation and can choose which pathway or activities they wish to complete.

All participants allocated to BrighterSide are provided by the study team with a smartphone with the BrighterSide app installed and the app is activated by the participant with an access code provided by the study team as part of onboarding. Onboarding and offboarding is by a study AP or other non-blind member of the trial team. Access to BrighterSide app is exclusively for use in a clinical trial and access is removed on exit from the study. Participants can email themselves a copy of their safety plan from within the app at any time and at offboarding. BrighterSide collects usage data that facilitates monitoring of fidelity and adherence with the intervention. Adherence is defined as engagement with at least two of the five modules.

There are no criteria for discontinuing or modifying allocated interventions.

### Training and supervision

PSWs require a minimum of at least 1-year experience as a PSW and to have completed a formal PS qualification/course. APs are required to have a bachelor’s degree in psychology or another related discipline and experience of working in a health and social care setting. APs and PSWs are NHS employed (either substantive or honorary contracts); receive training in the intervention manual and NHS mandatory training (including safeguarding vulnerable adults) and receive weekly supervision from a clinical psychologist that covers clinical issues and fidelity. PSW and APs are required to role play delivery of interventions to a satisfactory/competent standard prior to delivery to trial participants. To ensure ongoing reflective learning, consistency across sites and fidelity to the manual APs and PSW attend an intervention specific, group supervision. Both SAFETEL and PREVAIL involve safely addressing suicidal crises and participants are ask about suicidal thoughts or behaviours at each encounter. If endorsed, the AP or PSWs use a scripted risk formulation assessment algorithm to gather additional information regarding any recent suicidal behaviours, whether suicidal ideation has worsened since the thoughts were last discussed with a clinician and the person’s level of intent to act upon their thoughts. If any of these risk factors are present, the AP or PSW immediately contacts the mental health clinician on-call for the RAPID study to review the assessment with the patient still present, and it would be the clinician’s responsibility to determine the necessary next steps to ensure safety.

### Strategies to improve intervention fidelity and adherence

For both SAFETEL and PREVAIL, fidelity to delivery is checked by listening to audio recordings of the intervention sessions. Feedback is given to staff with opportunities for further refresher training if required. For SAFETEL, 20% of the initial safety planning sessions for which we have recordings will be checked for fidelity against a standardised rating scale of fidelity [20]. These will be double coded by another team member and tested for inter-rater reliability. Adherence to SAFETEL is defined as attending the initial safety planning session and at least one follow-up call as this was the definition of adherence provided by the developers of SAFEEL in their pilot work. For PREVAIL, fidelity will be monitored the fidelity checklist to ensure the conversation structure of Invite, Learn, Share and Motivate (ILSM) [25]. Adherence to PREVAIL is defined as attending at least PREVAIL sessions.

### Relevant concomitant care permitted or prohibited during the trial

Our participants are patients, whom at consent, are under the care of a community mental health team and/or have recently been under the care of a crisis team, which typically includes some or all the following: provision of care coordination from a Community Psychiatric Nurse, Social Worker or Occupational Therapist, outpatient psychiatry and access to psychology in accordance. In England, this delivery is in line with the National Institute for Health and Care Excellence (NICE) guidelines and in Scotland access to psychological interventions is in line with Matrix Guidelines relevant for the participant’s mental health condition. As it would be unethical to restrict access to treatment as usual for our participants, we do not prohibit any of the care provided by the treatment as usual services for the different diagnostic groups, so there is no single active comparator that would be suitable.

### Provisions for post-trial care

There is no provision for post-trial care from the research team. Participants have access to TAU, which as outlined is access to a secondary care community mental health service, or if discharged from secondary care they have access to primary care support including a General Practitioner and the national UK NHS helpline 111. All participants are offered a helpline card with local and national statutory (NHS) and non-statutory (voluntary) services that can offer support in a crisis. This is offered throughout the trial and on exit.

### Outcomes

#### Primary outcome

The primary outcome is any admission to a psychiatric hospital admission over 6 months (treated as a dichotomous yes/no variable) which is obtained by screening the participants’ electronic patient records (EPR).

#### Secondary outcomes

The secondary outcomes listed are collected by RAs at baseline, 3-month and 6-month assessment:

1. Suicidal thoughts and behaviours are assessed using the Columbia-Suicide Severity Rating Scale (CSSRS), which is a valid and reliable interviewer rated measure [32, 33]. As the CSSRS is an interview-based measure with assessors rating participant responses for each CSSRS item, we require all assessors to undergo training in delivery of the interview and scoring is checked for reliability against a gold standard provided by an expert assessor (ROC). Training for the CSSRS is provided by an expert in the field of suicide intervention research (ROC) and all RAs are required to complete two CSSRS role plays with the trial management team to ensure proficiency of clinical skills (warmth, non-judgmental stance, active listening) and skill in asking the required questions as per the CSSRS interview schedule.
2. Personal recovery using a service-user defined self-report measure of recovery, The Process of Recovery Questionnaire [34].
3. Health status using the EQ-5D-5L self-report measure, which is a health status questionnaire shown to have acceptable validity in people with schizophrenia in European countries [35].
4. The Recovering Quality of Life (ReQoL-10) self-report measure, which focuses on aspects of recovery and quality of life and was designed for use in a broad range of mental health conditions. It was collaboratively developed with service users and clinicians [36].
5. Wider NHS and social care use will be assessed by the Economic Patient Questionnaire, which is conducted in an interview format and completed from the participants self-report regarding use of NHS and social care services. It also includes the Psychiatric Hospital Record, completed from the EPR [37].
6. Anxiety will be measured using the GAD-7 a self-report measure [38].
7. Depression will be measured using the PHQ-9 a self-report measure [39].
8. Hope will be measured using the Adult HOPE Scale, which is an 8-item self-report questionnaire [40].
9. Entrapment will be measured using the 4-item self-report Entrapment Scale Short-Form [41].
10. Self-reported potential adverse effects of study participation measures on exit from the study at 6-month assessment [42].

We record and report all adverse events (AEs) and serious adverse events (SAEs) for each participant for the 6-month period that they are enrolled in the study. We are made aware of AEs and SAEs though the following sources: self-report from the participants, report from the participants care team and reports documented in the EPR and identified during the EPR screening for the primary outcome. Full details of AE and SAE definitions and reporting can be found below.

### Participant timeline

A time schedule of enrolment, interventions, assessments and visits for participants are in Table 1.

**Table 1:** Schedule of schedule of enrolment, interventions and assessments

**Time point**	**Enrolment** **−t1**	**Allocation** **0**	**Post-allocation**					
			**Month 1**	**Month 2**	**Month 3**	**Month 4**	**Month 5**	**Month 6**
**Enrolment**								
Informed consent	**X**							
Baseline assessment	**X**							
Allocation		**X**						
**Interventions**								
PREVAIL			**X**	**X**	**X**			
SAFETEL			**X**	**X**	**X**			
BrighterSide			**X**	**X**	**x**			
**Assessments**								
PHR	**X**				**X**			**X**
CSSRS	**X**				**X**			**X**
QPR	**X**				**X**			**X**
EQ-5D-5L	**X**				**X**			**X**
ReQoL-10	**X**				**X**			**X**
EPQ	**X**				**X**			**X**
GAD-7	**X**				**X**			**X**
PHQ-9	**X**				**X**			**X**
AHS	**X**				**X**			**X**
ESSF	**X**				**X**			**X**
Learning from You								**X**
Adverse events^a^								**X**

*AHS* Adult Hope Scale, *CSSRS* Columbia-Suicide Severity Rating Scale, *EPQ* Economic Patient Questionnaire, *EQ-5D-5L* Health Status, *ESSF* Entrapment Scale Short-Form, *GAD-7* General Anxiety Disorder Scale 7-item, *PHR* Psychiatric Hospital Record, *PHQ-9* Patient Health Questionnaire 9-item, *QPR* The Process of Recovery Questionnaire, *ReQoL-10* The Recovering Quality of Life Questionnaire

^a^Adverse events will be monitored at each time point and at every intervention session

### Sample size

We will randomise a maximum of 1064 participants (approximately 335 Northeast London; 224 for Manchester, East London and Glasgow; and 111 for Oxford); a total study sample of 1118 participants including the 54 participants allocated to BrighterSide arm. We power for an expected difference in proportion of admission rates between arms of 7.5%, and an expected admission rate within 6 months in the TAU group of 15% (based on extensive audit data from our NHS Trusts). The design follows the methods outlined for MAMS designs with binary outcomes [29] calculated using nstagebin in Stata. It uses the same outcome at both stages and has a primary endpoint of 6 months after recruitment in each stage and the loss to follow-up rate is 5%. One-sided significance levels of 30% and 2.5% and powers of 92% and 90% are used in the 1st and 2nd stages respectively. The total sample size of 1064 represents the maximum possible sample size under the 2-stage MAMS trial design: the minimum sample size if both arms are dropped for futility at the interim analysis would be 684. The interim analysis will take place after 385 participants provide 6-month outcome data, which will occur in May 2024. The interim analysis may result in one or both interventions being removed from the trial at stage 1 (power = 92%, one-sided alpha = 0.3). The final analysis has 90% power and a one-sided alpha = 0.025. The overall family-wise error rate (FWER) is 0.0397. There is no need to account for multiple testing in a multi-arm trial with independent comparators [43]. We have allowed for a 5% missing data in the primary outcome (this is routinely available in electronic medical records, so we should have less than 5% attrition which are likely to be withdrawals).

### Recruitment

We use multiple methods to ensure our recruitment strategy has maximum engagement with the relevant clinical services. The initial approach to these services is typically made by the PIs through established links at the site and where required the PIs have engaged with the services to support and problem solve any challenges with recruitment. Each site has two RAs who are embedded within the crisis teams, with weekly/bi-weekly attendance at the service team meetings sustaining a study presence and facilitating participant identification. Furthermore, this approach builds a positive relationship between the clinical staff and research team. To ensure ongoing engagement, the research team provides educational presentations regarding the research, as well as a regular supply of study leaflets and posters to the crisis teams. We also provide regular updates through study newsletters. Our recruitment approach and presence within the crisis services ensures fair access to all potential participants throughout the lifetime of recruitment by ensuring the crisis teams have confidence in the study design and team. We work closely with the National Institute for Health and Care Research (NIHR) Clinical Research Network and NHS Research Scotland Mental Health Network. Where in place, we utilise local NHS Trust standard operating procedures (SOP) for delegation of screening and first contact from service staff to the RDT.

Eligible participants are identified by a clinician from their crisis team or another individual delegated responsibility to identify potential participants by the crisis team leader. The initial approach to introduce the study and ascertain if the patient wants to proceed to be referred to a member of the study team is made by the crisis team clinician or delegated by the crisis team leader to another individual responsible for first approach. Following receipt of a referral, the site RAs contact the potential participant to introduce the study, answer preliminary questions and should the potential participant wish to arrange an informed consent visit (ICV) they are sent the PIS ahead of the ICV with at least 24 h to consider the information.

## Assignment of interventions: allocation

### Sequence generation

Randomisation (at the individual level) is independent and concealed using randomised-permuted blocks of random size administered by the King’s Clinical Trials Unit (KCTU; UK Clinical Research Collaboration registration 053) and which are not known to the study team. Randomisation is stratified by site. We use a 2:2:3 allocation in favour of TAU. In stage 2, we will keep the existing allocation or switch to 1:1 allocation if there is only one active intervention arm.

### Concealment mechanism

Randomisation is implemented via a study-specific web-based portal developed by KCTU. On randomisation the allocation is made known to the interventionists, the trial managers to monitor adherence to the randomisation algorithm and assign interventions to cases, the PI to ensure site oversight and the Trial Administrator who is delegated the duty of posting the allocation letter. Blinding of the allocation code of a subject is maintained for RAs until all outcome measures for that participant have been collected. All staff are required to follow a standard operating procedure for maintaining concealment of allocation and report of any accidental unblindings of the allocation.

### Implementation

Following informed and written consent, eligible participants are randomised within 2 working days by RAs blind to allocation using the study-specific web-based portal developed by the KCTU.

## Assignment of interventions: blinding

### Who will be blinded

RAPID is a single-blind RCT and as such trial participants are not blinded to assignment, since this would be impossible given the nature of the psychosocial interventions. For ethical and clinical reasons, we do not withhold the outcome of allocation from care providers given trial participants are likely to experience regular suicidal crises and study interventionists are required to share with the care provider any risks to self or others reported. Our primary outcome is objective (admission to hospital (yes/no)), so should not be subject to rater bias. RAs are blind to treatment condition. Blindness is maintained using a wide range of measures set out in our SOP for blinding including separate offices for the PSWs and APs and RAs, protocols for reminding crisis team clinicians, participants and family members about the blind and data file security of randomisation information. Blind breaks are recorded by the Trial Manager and reviewed by the Chief Investigator for patterns. When a blind break does occur, where possible, we will identify an independent assessor with whom the blind has not been broken to complete subsequent follow-ups, subject to any threats to participant engagement with follow-up. The trial statistician will remain blind to treatment allocation until the SAP has been finalised and approved; the senior trial statistician will remain blind until all analyses have been completed. Interim analysis of the primary outcome (admissions) will be carried out by the unblinded trial statistician and checked by an independent blinded trial statistician. Final analyses will be carried out by the trial statistician and overseen by the senior trial statistician.

### Procedure for unblinding if needed

There are no conditions under which the blind will be purposively broken.

## Data collection and management

### Plans to promote participant retention and complete follow-up

Our primary outcome of admissions is collected from routine electronic patient records (EPR) and so we expect minimal missing data. At point of consent, potential participants are informed that should they wish to change their status in the study they can choose from a number of options that includes dropping out of the intervention only, dropping out of the intervention (if allocated) and/or the research assessments whilst continuing consent to the primary outcome data collection from the EPR, or withdraw from all aspects of the study including primary outcome data collection via the EPR. Should a participant choose to withdraw from all research procedures we will not collect any outcome data from the point of withdrawal but will retain the data we have collected to the point of withdrawal. The choice in change of status provides maximum opportunity to continue to collect the primary outcome data. We allow for a small amount of consent withdrawal and other factors at a conservative level of 5%.

For the secondary clinical measures, a 20% loss to follow-up is approaching the upper limit beyond which the validity of trial findings would be jeopardised. To best support our participants and promote retention to follow-up, the RAs are trained to work in a person-centred manner where the participant is given as much control as possible over the method for the assessment (in-person or remote), location including option for a home visit or in a community centre, and time and date of the assessment. If a potential participant is unable to participate due to lack of a telephone/mobile phone, then we provide them with a mobile phone and 6-month data package. For ethical reasons, if we provide a participant with a mobile phone, we will allow them to keep it after they exit the trial to prevent reinstating digital exclusion and digital poverty for those that required a phone. Data are collected at three time points: baseline, 3-month and 6-month assessments. We have carefully selected the research assessment time points to ensure we can address the research questions, whilst balancing this with minimising participant burden. The assessment measures are clearly prioritised so that the most important will be collected first to minimise missing data and manage participant burden. RAs follow a distress protocol; this includes the option of a telephone contact within two working days of the assessments to provide a space for structured space to feedback on the assessment process and ask any questions about the process that may have arisen after the meeting. Participants are compensated for their time per assessment with £10 in cash, voucher or bank transfer. These processes are consistent with systematic review evidence for increasing retention in clinical trials [44]. To ensure continued oversight and support for RAs with follow-up retention, the RAs received weekly trial management supervision that includes monitoring of follow-up rate retention and proactive problem-solving for issues with completing assessments.

### Data management

All participant data are pseudonymised using a unique trial identification number allocated at enrolment, with data always kept securely at NHS and University bases in accordance with the requirements of GDPR. Paper source data worksheets are completed at sites and transferred to a web-based electronic data capture system backed by an electronic data management system (InferMed MACRO version 4; KCTU) that is hosted on a dedicated server within KCTU. The system is compliant with Good Clinical Practice. Roles are assigned to users, giving the ability to enter data relating to participants or to view data and raise discrepancies. A full audit trail of data entry and any subsequent changes to entered data is automatically date and time stamped, alongside information about the user making the entry/changes within the system. No data is entered onto the system unless a participant has signed a consent form to participate in the trial. The system is programmed to perform validation checks, such as range checks to prevent data entry errors. Missing data codes are programmed into fields, for ease of analysis. The system is also programmed to flag up when a missing data code is entered, to aid monitoring. A standard feature of InferMed MACRO data entry system is the built-in audit trail on all data fields, the automatic saving of data as you leave a form, and the ability to maintain a record of ‘source data verification’ checks. No data are amended independently of the study site responsible for entering the data. Data entered from paper source worksheets completed at sites will be checked against the electronic data for accuracy. Accuracy will be checked for 100% of the primary outcome on the post-baseline time points across all sites. If the error rate is greater than 1%, accuracy checks for all data will be triggered.

### Confidentiality

Participant data are strictly confidential, and all personally identifiable information (PID) is securely stored on NHS or University drives with access granted only to the research team with password protection. Paper forms for questionnaires are pseudo-anonymised with a Participant Identification Number (PIN) and are secured in locked NHS or University spaces separate to PID. Participants are made aware of the security and confidentiality of the data including the mandated limits to confidentiality when the study team is provided with information that indicates the participant or another person is deemed to be a risk to self or others. Audio recordings are carried out in line with the sites NHS policies and procedures. Audio recordings are transferred to a secure NHS drive and are only accessible by members of the research team with delegated responsibility to access, as per the study delegation log.

## Statistical methods

### Statistical methods for primary and secondary outcomes

A detailed statistical analysis plan will be prepared and approved by the Trial Management Group (TMG), Data Monitoring Committee (DMC) and TSC prior to the interim analysis.

Descriptive statistics within each randomised group will be presented for baseline values. These will include counts and percentages for binary and categorical variables, and means and standard deviations, or medians with lower and upper quartiles, for continuous variables, along with minimum and maximum values and counts of missing values. There will be no tests of statistical significance or confidence intervals for differences between randomised groups on any baseline variable.

For the analysis of the primary outcome at both stages, we will use a generalised linear model that includes fixed effects for treatment, diagnosis and site using maximum likelihood estimation. For each pairwise comparison with TAU, we will report the difference in proportion of admissions as the estimate of treatment effect with 2-sided 95% confidence intervals and 1-sided *p* values.

Secondary outcomes with repeated measures at 3 and 6 months will be analysed at stage 2 only. These will be analysed with generalised linear mixed models (GLMMs) appropriate for the distribution of the outcome using maximum likelihood estimation. Fixed effects will be treatment, diagnosis, site, time and time*group interactions, with a random effect for participant. Treatment effect estimates will be reported as adjusted between-group mean differences in outcomes between the groups separately at 3 and 6 months with 2-sided 95% confidence intervals and 1-sided *p* values.

### Interim analyses

For stage 1 interim analysis, only the primary outcome will be formally analysed using the generalised linear model described above. We designed the trial to have a non-binding futility bound with a one-sided *p* value of 0.3. The significance level will be used as the critical value for the *p* value of the observed treatment effect as this controls the type I error rate at the nominal level regardless of the true control event rate [45].

The measures to safeguard the confidentiality of the interim analysis results and minimise potential operational bias are detailed below:

- The independent DMC will receive a closed report prepared by the unblinded trial statistician showing the between-group differences of each intervention arm compared with TAU.
- If both intervention arms meet the criteria to continue in stage 2 (one-sided *p* value ≤ 0.3), no interim analysis results will be shared with the TMG or TSC, and the study will continue as three-arm trial, provided this is endorsed by the TSC on the recommendation of the DMC.
- If one or both interventions arms just exceeds the threshold for futility (one-sided *p* value > 0.3 and < 0.4), we will provide the DMC with additional information about the between-group differences on the CSSRS to guide a decision on whether one or both arms should continue in stage 2.
- If one intervention arm clearly exceeds the threshold for futility (one-sided *p* value ≥ 0.4), or if following the additional information on CSSRS the DMC recommends dropping one arm, the interim analysis results for this comparison only will be shared with the TMG and TSC on the recommendation of the DMC. This arm will be dropped from stage 2 of the study. The interim analysis results of the remaining arm will not be shared with the TMG or TSC.
- If both arms are recommended to be dropped, following confirmation of the recommendation of the TSC by the funder and sponsor, we will terminate recruitment to the study for lack-of-benefit in either arm.

### Methods for additional analyses (e.g. subgroup analyses)

There is no pre-specified subgroup analysis.

### Methods in analysis to handle protocol non-adherence and any statistical methods to handle missing data

All analyses, both interim and final analyses, will be conducted on the intention-to-treat (ITT) sample (i.e. all randomised will be analysed according to which study arm they have been allocated, regardless of the intervention or treatments actually received, adherence to treatment, or the number of measurements recorded).

The per-protocol population (PPP) will be defined as all participants that adhered to the interventions’ protocols, and participants that are classified as non-adherers will be removed from the PPP sample. Adherence is defined as follows for the treatment arms:

- SAFETEL: attending the initial safety planning session and at least one follow-up call
- PREVAIL: attending at least two peer support sessions

Analyses for the primary outcome (hospital admissions) will be repeated on the PPP sample as supplementary analyses at stage 2 only. If the proportion of adherence for any of the interventions is found to be lower than 80%, a complier average causal effect (CACE) analysis will be considered to investigate the effect of actually receiving the intervention on the primary outcome.

Maximum likelihood estimation provides valid inferences under the assumption that the missing data mechanism is ignorable (or missing at random). We anticipate low levels of attrition for the primary outcome (< 5%). We will check for baseline predictors of missingness in the secondary outcomes at 6 months and if identified include these in the analysis model as a sensitivity analysis.

### Plans to give access to the full protocol, participant-level data and statistical code

These will be managed and held by the King’s CTU. Requests for access to the dataset and analysis code will be considered in the first instance by the Chief Investigator and then the King’s CTU.

## Health economics and cost-effectiveness methods

A within trial economic analysis will compare the net costs and health benefits of the included interventions (PREVAIL and SAFETEL) plus TAU to TAU alone over the 6-month trial follow-up, from the NHS and social care (costs) and patient (health benefits) perspective. The primary measure of health benefit will be quality-adjusted life-years (QALYs) estimated from the EQ-5D-5L and the utility tariffs recommended by NICE at the time of the analysis. Health and social care service use (self-report supplemented by electronic medical record review for psychiatric hospital admission data) and EQ-5D-5L data will be collected for each participant at baseline, 3 months and 6 months. Self-reported service use data will be collected using an Economic Patient Questionnaire (EPQ) developed and used by the applicants in previous trials in severe mental illness and adapted for this study. The UK literature recognises that issues with electronic routine data sources may result in self-reported data being the preferred option [46]. Due to the unfeasible nature of accessing and extracting full-service use from routine electronic data sources, the trial will collect psychiatric inpatient admissions from hospital record data (these are likely a key driver of costs in this population). With the rest being taken from the self-report questionnaire (EPQ), collected during interview. Items of resource use will be multiplied by published national health and social care costs (Department of Health Reference Costs; Unit Costs of Health and Social Care, PSSRU). The EPQ will be discussed and piloted with the SURG prior to being finalised for the start of the trial.

Multiple imputation will be used to impute missing observations. The analyses will control for key baseline covariates or characteristics (demographic, socio-economic and clinical measures) identified from the published literature and supplemented with analysis of pooled baseline data. The cost and outcome effects will be bootstrapped to generate incremental cost-effectiveness ratios, cost-effectiveness acceptability curves and net benefit statistics. Cost-effectiveness acceptability curves (CEACs) will be plotted to summarise uncertainty associated with the ICER. To derive CEACs, the incremental cost and QALY (effect) estimates from the regression analyses will be bootstrapped to simulate the sample data of costs and QALY. The bootstrapped estimates of net QALYs will be revalued, using a range of ceiling ratios or willingness to pay thresholds (WTPT) to gain 1 QALY.

In the UK, there is no universally agreed cost-effectiveness threshold value. One commonly reported threshold is from NICE in England of approximately £20,000 to £30,000 per QALY [37]. However, there is a lack of consensus around the appropriate threshold [47, 48]. Therefore, the monetary value of simulated QALYs will be varied from £0 to £30,000 to reflect a range of hypothetical willingness to pay thresholds. As the analysis is comparing multiple options, a sensitivity analysis will adopt a fully incremental approach. Sensitivity analyses will also assess the choice of health benefit measure (QALYs calculated using ReQOL-10 as an alternative measure) and the impact of missing data (complete case versus imputed data). All economic analyses will be pre-specified in a Health Economic Analysis Plan (HEAP) to be agreed with the TSC and DMC. Reporting will be in line with the Consolidated Health Economic Evaluation Reporting Standards 2022 (CHEERS 2022) statement [49].

## Oversight and monitoring

### Composition of the coordinating centre and trial steering committee

The University of Manchester is the primary sponsor for the study. The day-to-day running and organisation of the RAPID trial is coordinated by a central management team comprised of the Chief Investigator (CI), a Trial Manager and a Clinical Trial Manager, and the site management teams that are comprised of a Principal Investigator (PI), site leads/coordinators and local clinical supervisors (where this function is not provided by the PI). The central management team meets on a weekly basis, and an extended meeting each month to ensure oversight of the study and coordinates a monthly Trial Management Group (TMG) meeting attended by the central management, site management teams, co-investigators and the sponsor. The site management teams meet with the RAs and interventionists for a weekly team meeting to ensure there is regular communication and interaction between site leads, local clinicians and research staff. One member of the central management team attends the site team meetings at a minimum of once per month to ensure central management presence and support. Each staff group receives the following: (1) local clinical supervision for personal wellbeing, risk management, problem solving local issues and compliance with local NHS policy; (2) a weekly/bi-weekly central management supervision to ensure compliance to the study protocol, reliability in conducting research assessments or fidelity to the intervention manual; and (3) a weekly/bi-weekly group supervision with their peers (chaired by a member of the central management team) to ensure across site peer connection/support, learning and consistency.

Independent oversight of the trial is provided by an independent DMC and a TSC. Full details of the DMC are provided below. The TSC is comprised of an independent chairperson, a statistician with expertise in MAMS design and analysis, two clinicians with expertise in SMHP and inpatient settings, and a service user along with non-independent members; the CI, trial manager, a representative of the funder and a representative of the sponsor are invited to attend. The TSC meet every 6 months and the meetings occur around 2 weeks after the DMC to ensure all the DMC recommendations are considered by the TSC. The funder is provided with the minutes of the DMC and TSC meetings along with a summary of the committee’s recommendations and actions taken. Both the DMC and TSC approved the study protocol ahead of the study commencing recruitment and review the internal pilot data against the a priori progression criteria with recommendations for the funder. The DMC and TSC shall review the interim analysis data; however, only the DMC shall have sight of unblinded data.

### Composition of the data monitoring committee, its role and reporting structure

The composition of the DMC is an independent chairperson, a statistician with expertise in MAMS design and analysis, and a clinician with expertise in serious mental health problems and inpatient settings. To facilitate the meeting, the CI, Trial Manager and study statisticians attend the DMC to facilitate the meeting and the sponsor invited to attend. The DMC meets six monthly, but major trial issues can deal with between meetings, by phone or by email. An open and closed format is used for the meeting and only independent members of the DMC and the unblinded trial statistician have access to unblinded data. For the open sessions, the meeting focusses on recruitment, retention, data quality (e.g. data return rates, treatment compliance) and adverse/serious adverse events. The closed session focuses on safety data by treatment group and selected secondary outcomes the randomised groups are labelled A and B, with the identity of A and B provided by the study statistician separately to the report. A copy of the DMC charter is retained by the trial manager in the site file.

### Adverse event reporting and harms

An adverse event (AE) is defined as any untoward medical occurrence, unintended disease or injury or any untoward clinical signs (including an abnormal laboratory finding) in subjects, users or other persons whether or not related to the research procedures or intervention. This definition includes events related to the study interventions and events related to the procedures involved. A serious adverse event (SAE) is defined as an adverse event that: (1) results in death, (2) is life-threatening, (3) requires hospitalisation or prolongation of existing hospitalisation, (4) results in persistent or significant disability or incapacity, (5) consists of a congenital anomaly or birth defect, or (6) is another important medical event if determined to be serious based on medical judgement. Data on adverse and serious adverse events will be collected via three sources (1) participant self-report (to a study team member), (2) identification through EPR screening for the primary outcome data or risk updates ahead of assessments or intervention sessions, and (3) we may become party to an adverse event during contact with a participants care team. Study team members are required to report all adverse and serious adverse events in line with our trial standard operation procedure for AEs for all participants from the time of their enrolment into the study as defined as the time at which a participant sign and dates the informed consent form.

AEs and SAEs are reviewed by the study CI or another delegated clinician to determine severity, intensity, causality and expectedness. Thoughts of suicide are associated with increased rates of suicide [50] and between 25 and 58% of people who report suicidal ideation will make a suicide attempt amongst those with suicidal ideation, between 25 and 58% will make a suicide attempt [51]. In line with previous trials, expected adverse events include worsening of symptoms defined as an increase in severe suicidal thoughts or a suicide attempt [24].

To ensure independent scrutiny of all AEs and SAEs, we report to several oversight groups: SAEs are reported to the sponsor every 2 weeks. For the period the trial was determined to be a clinical trial of a medical device all SAEs were reported to the MHRA and to the sponsor within 3 working days. All adverse events and serious adverse events are summarised in a report to the sponsor every 3 months. AEs and SAEs are reported to the DMC at each meeting. The study follows mandated reporting to the Health Research Authority (HRA). Reportable events which indicate a risk of imminent death, serious injury or serious illness and require prompt remedial action for other participants must be reported immediately and no later than 2 calendar days from sponsor awareness of the event. SAEs which are both related and unexpected must be reported immediately and no later than 15 calendar days from sponsor or CI awareness of the event.

We administer a measure of potential adverse effects from trial involvement at point of exit [42].

### Frequency and plans for auditing trial conduct

Audit of trial conduct is carried out by the sponsor and delegated to the study trial managers in line with a sponsor-approved monitoring plan. The sponsor conducts the audit of the Trial Master File. Site audit is conducted by the delegated Trial Managers and includes monitoring of the Investigator Site File in full for completeness, accuracy and completion of eligibility check forms, accurate and complete recording of participant change of status, informed consent procedures in line with the protocol and GCP including evidence of at least 24 h to consider the study information, accuracy of informed consent forms and consent taken only by those delegated as per the delegation log, secure and confidential storage of participant data. Audit is not independent of the investigators and the sponsor.

### Plans for communicating important protocol amendments to relevant parties (e.g. trial participants, ethical committees)

The funder and the sponsor approve important protocol amendments. An opinion is sought from the DMC and TSC for amendments when the funder deems it sufficiently required. All amendments are summarised in the DMC and TSC report provided ahead of these meetings. The RAPID trial is required to follow UK Health Research Authority (HRA) regulation and procedures for amendments using the HRA amendment toolkit, which categorises amendments into non-substantial or substantial and determines a category regarding extent of approval required from study sites. The amendment toolkit is the vehicle for communicating and obtaining approval for amendments from the REC, HRA and sites where required. Prior to November 23, 2023, all substantial amendments required MHRA review and approval, which was requested by email communication with the MHRA following their requirements. Important amendments are updated on the trial registry by the Trial Manager.

## Dissemination plans

The results from this study will be published in a peer-reviewed journal for dissemination amongst researchers and clinicians. Results of the study will also be disseminated to trial participants. Patient and Public Involvement will be central to producing summaries of the research finding to ensure accessibility. The results will be disseminated amongst healthcare professionals and the crisis teams that have helped support the trial. We will develop guidance and policy briefings for policy makers and commissioners. The training materials and intervention manuals will be made freely available via a web portal.

## Discussion

We have presented a protocol for an adaptive RCT of three brief psychosocial interventions for people with a SMHP, who have had a recent suicidal crisis and crisis care. Our study is highly novel as it is the first trial that we are aware of that uses an adaptive design to investigate interventions to reduce avoidable psychiatric hospital admissions by targeting suicidal ideation and behaviours. Adaptive designs are flexible and efficient approaches to studying multiple interventions [29] and the RAPID trial will provide evidence from a single study regarding clinical and cost effectiveness of a range of interventions that are brief, accessible, and if effective, scalable within the healthcare system. This study will address several unmet needs including improving the efficacy and accessibility of psychosocial interventions, developing the workforce and responding to the aim of preventing avoidable hospital admissions. Furthermore, targeting the secondary outcomes of suicidal ideation and behaviour addresses an international call by the World Health Organisation to improve suicide prevention by upskilling the healthcare workforce and enable effective follow-up with people who have had a recent suicidal crisis [17].

We have taken a rigorous approach to minimise bias including the randomisation allocation generation via permuted blocks that is issued by a centralised web-based computer system hosted by a UK registered CTU, specific study operating procedures to minimise detection bias, pre-specification of statistical analyses, including the pre-planned interim analysis, and a rigorous approach to recording and reporting adverse and serious adverse events.

There are several challenges with this adaptive design that may also represent limitations. Removal of the BrighterSide arm was an unplanned change, so it is not possible to say conclusively whether the arm would have been dropped for futility in this population. However, the evidence from the definitive Australian trial was conclusive that it did not have any effect in a population that presented with less complexity in terms of mental health problems and, ethically, dropping it based on new evidence combined with our usage data was considered appropriate. Delivery of an adaptive design where the interventions are talking therapies delivered by staff requires careful management of the message regarding the purpose and planning for the outcome of the interim analysis to ensure staff wellbeing given the uncertainties created for contracts of employment. The treatment costs for delivering such interventions within the context of NHS research structures are not funded as part of the research and this presents a unique challenge when navigating cost attribution for an adaptive design. It can be complex and requires strong communication, understanding and commitment from stakeholders regarding the adaptive design process. Resources for trial management in studies where the intervention targets suicidality need careful consideration to ensure the study is well equipped to manage the potential high rates of adverse and serious adverse event reporting and strong clinical support for the management of active risk in trial participants. Our delivery staff (PSW and APs) and RAs are working within the context of risk that is often serious and challenging and within the context of mental health services which are seeing rising numbers of people accessing mental health services, with little growth in the mental health workforce to deliver these services [52]. As such, delivery of the trial requires a well-equipped and skilled workforce with clear systems and capacity to offer wellbeing support to minimise the risk of stress or burnout, and training to ensure a confident workforce. Our operating procedures for managing and escalating risk and a manualised approach to intervention delivery offer the required structure and support. Individual supervision and staff peer connection via regular group supervision provides space to discuss wellbeing, with an emphasis on celebrating examples of success or good practice as well as situations that have been challenging or complex. Group supervisions are a space to foster connection and build a support network for staff.

In summary, the majority of PHAs occur in response to a suicidal crisis. The PREVAIL and SAFETEL intervention both show promise regarding acceptability to service users and feasibility for further testing in a high quality, definitive trial. However, evidence about clinical effectiveness of these interventions to reduce PHAs is limited. If either intervention under investigation was shown to be superior to standard care, this would prove a major advance in follow-up care and treatment for people with SMHPs who have experienced a suicidal crisis.

## Trial status

Version 8.0 of the trial protocol (23/NOV/2023) has received ethical approval and is in the date of publication of this trial protocol. Recruitment commenced on August 1, 2022. Recruitment for stage 1 of the study is expected to continue until June 30, 2024. If the trial progresses to stage 2, recruitment will be completed by July 31, 2025.

### Supplementary Information

Additional file 1. SPIRIT checklist.

## Data Availability

The final trial dataset will be managed and held by our CTU, KCTU, and reasonable requests for access to the dataset will be considered in the first instance by the CI and then the CTU.

## Abbreviations

AE: Adverse event
AP: Assistant Psychologist
BIC: Brief intervention and contact
CACE: Complier average causal effect
CBT: Cognitive behaviour therapy
CEAC: Cost-effectiveness acceptability curves
CHEERS: Consolidated Health Economic Evaluation Reporting Standards
CI: Chief Investigator
CRN: Clinical Research Network
CSSRS: Columbia-Suicide Severity Rating Scale
CT: Cognitive therapy
CTU: Clinical Trials Unit
DBT: Dialectical behaviour therapy
DMC: Data Monitoring Committee
EPQ: Economic Patient Questionnaire
EPR: Electronic patient record
FWER: Family-wise error rate
GCP: Good Clinical Practice
HBTT: Home-Based Treatment Team
HRA: Health Research Authority
ICV: Informed consent visit
ITT: Intention to treat
LwDT: Living with Deadly Thoughts
MAMS: Multi-arm, multi-stage
MHRA: Medicines and Healthcare Products Regulatory Agency
MI: Motivational interviewing
NHS: National Health Service
PI: Principal Investigator
PID: Personally identifiable data
PIS: Participant Information Sheet
PHA: Psychiatric hospital admission
PPP: Per-protocol population
PS: Peer support
PSW: Peer Support Worker
QPR: The Process of Recovery Questionnaire
QALYs: Quality-adjusted life-years
RA: Research Assistant
RAPID: Remote Approaches to Psychosocial Intervention Delivery
RCT: Randomised controlled trial
REC: Research Ethics Committee
SAE: Serious adverse event
SMHP: Serious mental health problem
TAU: Treatment as usual
TMG: Trial Management Group
TSC: Trial Steering Committee
UK: United Kingdom
WTPT: Willingness to pay thresholds
